# KCl-induced repetitive cortical spreading depression inhibits trigeminal neuronal firing mediated by 5-HT1B/1D and opioid receptor

**DOI:** 10.1186/1129-2377-14-S1-P69

**Published:** 2013-02-21

**Authors:** W Supronsinchai, J Hoffmann, S Akermann, PJ Goadsby

**Affiliations:** 1 Headache Group, Department of Neurology, USA

## Background

Cortical spreading depression (CSD) is a wave of neuronal depolarization and follwed by suppression of activity, which has been implicated in the pathophysiology of migraine aura. It has been demonstrated that KCl-induced CSD can facilitate activity of trigeminocervical complex neurons. However, the mechanism of CSD-induced trigeminal neuronal activation is unclear.

## Objectives

To study the effects of repetitive CSD on the responses of trigeminocervical complex (TCC) afferents to nociceptive activation of the dura mater. In addition, to characterize the role of 5-HT and opioid receptors on CSD-induced changes in TCC activity.

## Methods

Adult male Sprague-Dawley rats were anesthetized with pentobarbitone sodium (60 mgkg-1). The parietal bone was removed over the MMA for placement of a bipolar stimulating electrode. For recording neuronal activity, a tungsten electrode was inserted into the TCC. CSD was induced by placing solid KCl (3 mg) on the parietal cortex.

## Results

A total of 30 neurons in the TCC responsive to nociceptive activation of the dura mater were studied. A sub-poplation, 9 out of the 30, showed an average inhibition of firing of 65±14% from baseline with CSD. This inhibitory response was reversed by intravenous administration of the 5-HT1B/1D receptor antagonist, GR127935 (3mg/kg), and a mu-opioid antagonist, naloxone (1.5 mg/kg), five minutes after injection (p < 0.05).

## Conclusion

The present findings show that repetitive CSDs inhibit a subpopulation of dural nociceptive trigeminal neurons, an effect mediated by serotonin and opioids receptors. Understanding how the cerebral cortex modulates trigeminovascular nociception will improve our understanding of the pathophysiology of migraine.

